# The Use of Split-Thickness Skin Grafts on Diabetic Foot Ulcerations: A Literature Review

**DOI:** 10.1155/2012/715273

**Published:** 2012-05-14

**Authors:** Brant McCartan, Thanh Dinh

**Affiliations:** Division of Podiatry, Beth Israel Deaconess Medical Center, 185 Pilgrim Road Baker 3, Boston, MA 02215, USA

## Abstract

Diabetic foot ulcerations are historically difficult to treat despite advanced therapeutic modalities. There are numerous modalities described in the literature ranging from noninvasive topical wound care to more invasive surgical procedures such as primary closure, skin flaps, and skin grafting. While skin grafting provides faster time to closure with a single treatment compared to traditional topical wound treatments, the potential risks of donor site morbidity and poor wound healing unique to the diabetic state have been cited as a contraindication to its widespread use. In order to garner clarity on this issue, a literature review was undertaken on the use of split-thickness skin grafts on diabetic foot ulcers. Search of electronic databases yielded four studies that reported split-thickness skin grafts as definitive means of closure. In addition, several other studies employed split-thickness skin grafts as an adjunct to a treatment that was only partially successful or used to fill in the donor site of another plastic surgery technique. When used as the primary closure on optimized diabetic foot ulcerations, split-thickness skin grafts are 78% successful at closing 90% of the wound by eight weeks.

## 1. Introduction

There are many means of treating diabetic ulcerations. A conservative approach may entail regular debridement and dressing changes. Topical solutions such as saline, iodine, antimicrobial absorbent fiber sheets, and collagenase ointments may be included. For wounds with macerated edges it may be adventitious to apply gauze with diluted iodine to prevent further maceration. In hyperkeratotic, fibrotic, or dry necrotic tissue borders, it is preferred to apply hydrogels to hydrate the surrounding area. It is important to debride fibrotic wounds. This can be done mechanically by applying saline wet to dry dressings, and then after the dressing is changed, it removes fibrotic tissue with it. Some institutions employ the use of negative pressure wound therapy (NPWT) to stimulate granulation tissue and help remove fibrotic tissue formation [1]. Also, NPWT is good for draining wounds, along with calcium alginates which help absorption. Collagenases can be used to chemically debride wounds, and sharp debridement is a time tested method to remove non-viable tissue. There are also several bioengineered products that may facilitate wound closure once the wound is infection-free and has a primarily granular base. These materials help deliver fibroblasts to wounds and help serve as a scaffold for new tissue growth. Sometimes chronic wounds remain or the wound is deep with an irregular contour, and plastic surgery techniques must be employed such as skin grafts and flaps. More advanced flaps are ideal for plantar or weight-bearing wounds because they have more substance and contain their own blood or nerve supply which increases graft take. These are also indicated in wounds with avascular bases such as directly over tendons, or bone without periosteum. Advanced techniques are challenging at times and create a new wound or leave a large donor site deficit. This, on a diabetic patient with diminished healing properties and increased susceptibility to infection, is not ideal. Thus, if a flap is not possible, split-thickness skin grafts may be the better treatment option to close challenging wounds once a granular base is achieved.

Split-thickness skin grafting (STSG) is a plastic surgery technique with documented use dating back to 3000 B.C. in India for traumatic facial wounds [2]. Ollier began experimenting with skin grafting methods in 1872. An electrodermatome was used to harvest STSG by Pagett & Hood in 1939 [3]. Modern techniques involving the use of meshed STSG was first described in 1964 by Tanner et al. [4]. Despite the original use for facial reconstruction, STSG is now commonly employed on burn wounds, when skin coverage is required, and to close chronic ulcerations, frequently seen in the diabetic population. Recent literature on burn patients has shown STSG to have a 96.7% graft take in optimal conditions [5].

STSG in diabetic wounds poses several unique concerns including the presence of neuropathy, endothelial dysfunction, and increased susceptibility to infection. Neuropathy is a concern because the patients are not in pain and are often unaware of the severity of their wound until the infection spreads more proximally. Endothelial dysfunction disrupts all vascular components especially the microvasculature seen in eyes, kidneys, and distal extremities. Also, hyperglycemia retards the chemotaxic response to fight infection. These systemic challenges, coupled with patient-specific obstacles that all surgeons entertain, namely, tobacco history, poor nutrition, and noncompliance, make the care of chronic wounds challenging. As a result, many surgeons select conservative care with regular dressing changes to treat diabetic wounds. When this fails, it is best to choose a plastic surgery modality that the surgeon is comfortable with and has minimal morbidity to the patient. This procedure should also be definitive and restore a healthy skin barrier. These authors prefer to use STSG to close challenging wounds because it causes little morbidity to the patient and its documented success in burn patients [5]. When STSG is utilized, the recipient wound bed needs to be granular taking care to remove all infected tissue and optimizing the vascular status prior to application. The purpose of this study was to analyze the literature to determine the indications and success of split-thickness skin grafts used on diabetic wounds and the reported risks and complications.

## 2. Materials and Methods

In an effort to determine the efficacy of this treatment, a literature review was undertaken to evaluate the outcomes of STSG in diabetic patients. These authors undertook a systematic review of electronic databases, namely the U.S. National Library of Medicine, National Institutes of Health-PubMed.gov (http://www.ncbi.nlm.nih.gov/pubmed/), from inception until 6/21/11. There was no restriction on data or language, and an inclusive text word query for “Diabetes” AND “Split-Thickness Skin Graft” was entered, where the upper-case words represented the Boolean operators employed. 

Forty articles were secured using this method. Following this, the references from the original forty articles were analyzed searching for the same inclusion criteria. After review, twenty-two articles were yielded that described STSG on diabetic patients. All articles pertained to lower-extremity wounds, though this was not detailed in the inclusion criteria of the search. A meta-analysis was then undertaken to find commonalities amongst the articles. These authors defined “success” to be 90% epithelization of the STSG recipient site by eight weeks using a single procedure with no infection or reulceration at final follow-up. Success rates were analyzed from the results of the four primary articles that specifically reviewed STSG on diabetic wounds [6–9].

## 3. Results and Discussion

Twenty-two articles were yielded from the initial search [6–27], with four articles found specifically addressing STSG on diabetic wounds [6–9]. These studies ranged from a case presentation to a prospective, randomized controlled trial and accounted for a total of 229 diabetic patients with lower-extremity wounds. 

Of the remaining eighteen articles, eight detailed techniques on application of STSG, or as one possible treatment option used on diabetic wounds [10–17]. Three articles used bioengineered or advanced techniques (platelet-rich plasma) in conjunction with STSG [18–20]. Three articles applied STSG over a (gracilis muscle) free flap or subcutaneous flap [21–23]. Two articles utilized STSG on top of a donor site to an advanced plastic technique: reverse sural artery flap and medial plantar artery flap [24, 25]. The last two employed STSG to heal a partially closed wound treated with another modality or to help close a burn site [26, 27].

The study purpose of the four articles describing STSG on diabetic wounds varied, as depicted in Table 1 [6–9]. Ramanujam et al. [8] retrospectively examined the success of STSG as a definitive treatment for wounds in patients with diabetes while Mahmoud et al. [6] prospectively compared the effect on healing times of STSG versus conservative wound dressing in the treatment of diabetic foot ulcers. The remaining two studies evaluated the influence of meshing the STSG [7] and the use of 10% phenytoin ointment as a wound bed preparation [9] prior to STSG application.

The etiology and location of the diabetic ulcers also varied significantly between the four studies. Three out of the four papers studied ulcers located on any area of the foot, including the plantar aspect, heel, interdigital area, and dorsum of the foot [6, 7, 9]. In addition to neuropathic diabetic foot ulcers, Ramanujam et al. included foot and ankle wounds stemming from traumatic, surgical, and infectious origins. All studies pertained to lower-extremity wounds and excluded an ulcer or wound with clinical signs of infection. It is not recommended to apply a STSG over infected tissue or exposed bone, tendon, or ligaments. This was not excluded in the research presented, but using a STSG over avascular tissues is contraindicated.

Mahmoud et al. studied the difference in days to heal and days spent in the hospital in 100 diabetic patients (fifty in each group) using STSG versus conservative wound care [6]. All the patients in the study did heal completely; however, the STSG group healed in an average of 28 days, versus 122 days in the conservative group. The mean hospital stay was also decreased by twelve days. Puttirutvong et al. assessed meshed versus nonmeshed skin grafts in eighty diabetic patients [7]. There was no statistical difference between the two groups, and again all patients in the study healed completely. Ramanujam et al. retrospectively analyzed STSG in 83 consecutive diabetic patients [8]. All of the patients healed successfully by the final follow-up, but this included repeat surgical procedures or additional graft applications. Younes et al. concluded that phenytoin ointment (originally used orally as an anticonvulsant) prior to application of STSG is safe and enhances the survival of the graft on sixteen diabetic patients [9].

Despite each article's different purpose and postoperative protocol, a meta-analysis was performed and found a similarity of STSG success. In Mahmoud's article, it mention that thirty-one patients had 100% graft take at week eight in the discussion. However, it also states that 86% (forty-three) patients had healed completely by week eight [6]. This author used the greater healing percentage (86%) in the table provided (Table 1), understanding that while graft take was 100%, only 86% healed by week eight. Puttirutvong used STSG on all the study patients; however, the control group consisted of 42 unmeshed grafts and 35 of these patients had “good” to “excellent” healing [7]. “Excellent” was described as skin grafts healed 95% within 2 weeks with a smooth scar; “good” was skin graft healed 95% within 3 weeks and a hypertrophic scar subsided within 6 months. The meshed group consisted of 38 patients; 31 of these had “good” to “excellent” healing. This offered a combined 82.5% “good” to “excellent” healing. Ramanujam's retrospective review showed 65% of patients healed uneventfully [8]. Younes' case study revealed 15 of 16 STSG patients had +90% healing with 2–8 weeks of preparation with phenytoin [9]. Combining these results in 229 patients, STSG is 78% successful, where success is described as 90% graft epitheliazation with a single procedure at eight weeks and no documented reulceration or infection to the recipient site at final follow-up.

The four primary articles in this review originated from four different countries [6–9]. Further analysis of the articles showed that graft size, donor and recipient location, patient age, gender, and race did not Effect graft survival per Ramanujam [8]. Most, (60%) of the patients in Mahmoud's study had a wound size between five and ten centimeters; there was no mention of wound location, depth, diabetic control, or vascular status [6]. Also, in the Puttirutvong study: depth, location, diabetic control, & vascular status were not addressed. The range of ulcer size was between 12 cm^2^ to 600 cm^2^ [7]. Younes only examined large ulcers, described as >20 cm^2^ [9]. It should also be noted that the articles did not mention the patients lower-extremity perfusion, which is an important factor of graft success, along with a well-prepared wound bed. Also, most articles failed to acknowledge the exact location where the graft was executed, and whether the graft was used on a weight-bearing surface, where greater substance flaps have a better survival. In our institution, a great deal of consideration goes to preparing the wound and off-loading the ulceration. This is also achieved by insuring that infected tissue or exposed bone (osteomyelitis) is removed. The patient's nutritional and vascular status is optimized if inadequate or when peripheral arterial disease is present. Once this is achieved, the wound is debrided until a healthy granular base is present. If the wound bed is deep or the contour uneven, we use NPWT to bring up an even, granular base. Following this, the patient is taken to the operating room for STSG application. Only Puttirutvong [7] and Ramanujam [8] presented their harvesting technique in detail. At our institution, a “classical approach” is utilized with a 0.015 inch thick setting on the dermatome used to harvest the graft from the lateral thigh or leg. We traditionally use local anesthetic with epinephrine in the underlying subcutaneous tissue and mineral oil at the donor site for ease of dermatome gliding. The donor site is covered with a nonadherent petroleum gauze dressing. Then, the graft usually meshed at a 1.5 or 3 : 1 ratio prior to application. There are some newer studies that use a polyurethane membrane over the donor site skin [28]. This membrane is also used on the recipient site instead of NPWT and has shown decreased operating room time, no suture requirement, and maintenance of STSG hydration.

Application of the skin graft, postoperative dressings and protocol differed amongst the authors, seen in [6–9] (Table 2). We prefer to use an absorbable suture to fix the graft suturing from the graft to the recipient site wound edges. Following this, we apply a nonadherent dressing atop the graft followed by NPWT typically set at 75 mmHg continuous pressure. The patient is made non-weight bearing to this extremity. The donor site dressing is removed in two days and kept dry. The VAC at the recipient site is taken down five days postoperatively. Next, a non-adherent dressing is placed over the STSG to keep it moist and prevent any shearing. The minimum follow-up was also not consistent in the research, ranging from six months to one year. We have found that the STSG becomes engrafted within two weeks and fully healed in about four weeks. There are several post-operative dressings that can be used with a bolster dressing: gauze, fibrin glue, silicone splints, foams, and other self-adherents. An important factor is immobilizing the graft to ensure capillary ingrowth during the first 2–5 days of inosculation [30]. This is difficult on the lower extremity and any area of uneven, irregular surfaces. Analyzing some of the literature, there has been several studies comparing the ideal dressing for both the recipient and donor site. Over eighty articles are accessible solely analyzing donor site dressings. Studying the most recent articles, no “best” donor site dressing exists: “the ideal dressing material for handling of the donor site is yet to be developed and extensive variability exists in the management of the STSG donor sites.” [29]. Ironically, the donor site is the primary cause of pain and distress for patients. Because of this, more research should be devoted to finding the ideal donor site dressing. Also, a study comparing the location of the donor site would be valuable; for instance, harvesting skin from a more distal site (lower leg or foot) in a patient with peripheral neuropathy may be less painful.

The articles in this literature review did not use a wound VAC to immobilize their STSG. Some more recent techniques [30–32] have shown success using a wound VAC to immobilize the graft during the inosculation period, upwards of 97% [1, 26]. NPWT provides uniform pressure over the entire grafted area. The pressure is usually set to be continuous, and normally between 75 mmHg to 125 mmHg. The VAC is then typically left in place four to five days.

**Figure 1:**
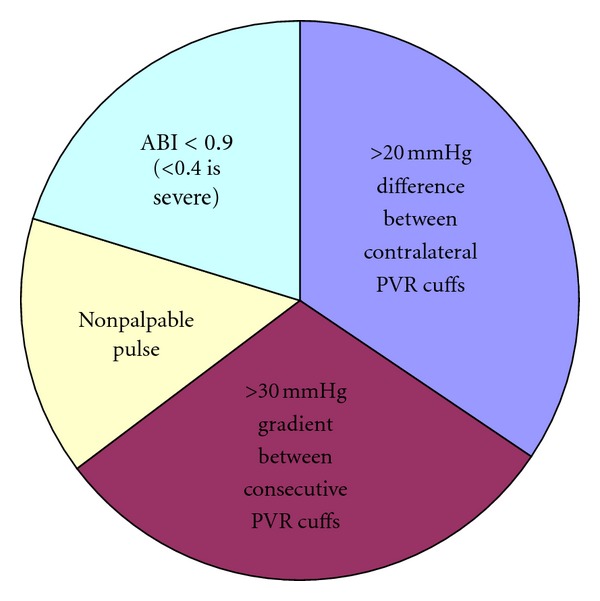
Indicators of peripheral artery disease.

It should be noted that none of the articles describe the importance of optimizing good vascular supply prior to application. The vascular status must be assessed, especially if a distal pulse—posterior tibial artery, perforating peroneal artery, or dorsal pedis artery is not palpable. If a triphasic wave form is not found with a hand-held doppler, non-invasive studies are recommended. These authors prefer to find the ankle brachial indexes (ABI) with pulse volume recordings (PVRs). The vessels media may be calcified (which can be seen on X-rays—Monckeberg's sign). This makes the vessel noncompressible or give an elevated ABI >1.0. If the ABI is less than 0.90, peripheral artery disease (PAD) is expected, as seen in Figure 1. If the ABI is less than 0.40, the disease is severe and vascular intervention is indicated. PVRs are helpful and complete the ABI exam. Plethysmography helps gauge the blood flow or perfusion to the limb without targeting a specific vessel. If there are inequalities between contralateral limbs (>20 mmHg), or if there is a large drop in pressure between adjacent cuffs on the ipsilateral limb (>30 mmHg), vascular occlusive disease is expected. If pressures are abnormal, and the waveform has a short and wide peak, extensive disease is present [33, 34]. If results are abnormal, a more invasive exam is recommended like angiography to determine the exact location and severity of the ischemia and potential revascular procedures.

**Figure 2:**
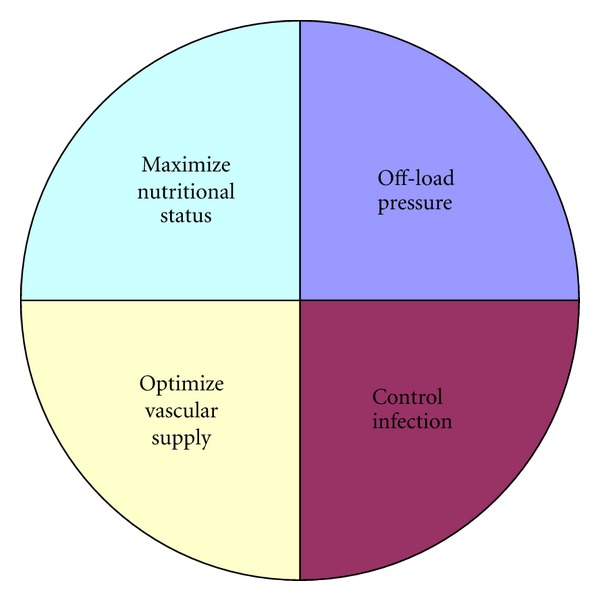
Principle factors in diabetic wound healing.

Along with optimizing vascular status, controlling infection and creating a healthy wound bed is pertinent prior to STSG application, as these two factors may be the primary reason why the diabetic wound is chronic. Wound bed preparation is a constantly evolving science with a focus on making a chronic wound acute. It is important to start with the basics: remove infected, nonviable tissue with regular debridements & ensure that the wound bed has good vascular supply to promote a healthy, moist wound bed and promote epithelial advancement. This is a dynamic process where all non-viable & infected tissues must be removed and antibiotic therapy is traditionally started. If osteomyelitis is suspected (there is exposed bone or the wound probes to bone), this must be addressed before the wound is closed. Two other factors to monitor, and which may cause the initial ulceration are excessive pressure and poor nutritional status.  Figure 2 depicts the principle factors to grant a successful graft and close a diabetic ulceration.

There are different processes to prepare the wound bed if the bodies natural response is lethargic. This institution prefers mechanical & surgical debridements prior to grafting because it is time tested and gives quick results. For patients with more ischemic wounds and those for which surgery may be less urgent, we turn towards enzymatic or collagen-based debridements to maintain more healthy tissue. Biologics & biosurgery are other methods that can be employed, though not routinely at this institution. There is increased interest in applying honey to wounds to help stimulate autolytic debridement [35]. Also, sterile maggots have been used over eighty years (1931) for successful wound debridement & preparation [36]. No matter the approach to preparing the wound bed, basic healing principles must also be ensured for graft survival and long-term viability.

## 4. Conclusions

On burn patients, split-thickness skin grafts have upwards of 100% success rates. There is minimal research available studying STSGs on diabetic patients. The majority of literature available on diabetic patients is retrospective reviews or case studies, which further limits this literature review. Also, the accessible articles do not mention the vascular status of the patients, which is often diminished in diabetics; this is an important factor in wound healing. Infection control, nutritional status, and pressure off-loading are pertinent to treating diabetic wounds and optimizing the recipient wound bed for STSG survival. Another aspect that was limited in the revises is the location of the wounds and graft application. Though wound size is included, depth and location are lacking in the articles. These factors help determine which plastic surgery technique can be utilized. There are better options than STSG for weight-bearing or plantar surfaces that can resist direct pressure and sheer forces. Also greater substance flaps should be employed atop bone and ligamentous and tendinous structures to prevent adhesions and ultimately graft failure, where STSG is contraindicated due to avascularity of these tissues. 

Despite these limitations, the accessible research [6–27] demonstrates that split-thickness skin grafting is versatile: it can be used as the primary modality on the recipient wound, secondarily as an adjunct to a partially accepted/failed treatment, or on the donor site of another plastic surgery modality. When used as the primary closure on optimized diabetic foot ulcerations, split-thickness skin grafts are 78% successful at closing 90% of the wound by eight weeks.

## Figures and Tables

**Table 1:** Meta-analysis of STSG on diabetic wounds.

Lead author	Study type	Purpose	Patients	Follow-up	% success*
Mahmoud (Sudan)	Nonrandomized case-controlled prospective comparative	Determine the difference in hospital days and days to heal between STSG and conservative care	50 (in each group)	1 year (following closure)	86%

Puttirutvong (Thailand)	Prospective randomized controlled	Assess meshed versus non-meshed skin grafts	80 (38 meshed)	6 months (from application)	82.50%

Ramanujam (USA)	Retrospective consecutive review	Analyze STSG in diabetic patients	83	>6 months (from application)	65%

Younes (Jordan)	Case study	Determine the impact of phenytoin ointment prior to STSG application	16	FU not mentioned	93.75%

*Success as determined by 90% graft epithelization by 8 wks with 1 procedure & no documented reulceration or infection to initial site at final follow-up.

Abbreviations: STSG, split-thickness skin graft; FU, follow-up; wks, weeks.

**Table 2:** Postoperative courses.

Lead author	Dressings to recipient site	Antibiotics	Postop Weight bearing	1st dressing change	FU appointments
Mahmoud	Paraffin gauze, diluted povidone-iodine- soaked gauze, sterile gauze and roll bandage	N/A	Off-loading as required	post-op day 5	5 days, 2 wks, 3 wks, 8 wks, monthly

Puttirutvong	Non-adhesive gauze, wet swab with NSS and mild pressure outer layer	N/A	Weight bearing status not detailed	post-op day 1	N/A

Ramanujam	Bolster dressing: sterile nonadherent petrolatum gauze, several sterile plain sponges moistened in saline attached with skin staples, short leg cast/posterior splint	>2 wks oral with + wound cultures	Non-weight bearing in short leg cast or posterior splint × 3-4 wks	3-4 wks post-op	every 2 wks x 2 months, every 3-4 months once healed

Younes	Sterile nitrofurazone dressing, gauze, backslap of plaster of Paris for 1-2 wks	N/A	Post-op splint × 1-2 wks	post-op day 3 or 4	N/A

Abbreviations: N/A, not applicable; postop, postoperative; FU, Follow-up wks; weeks, NSS, normal sterile saline.
